# Composites Based on Natural Zeolites and Green Materials for the Immobilization of Toxic Elements in Contaminated Soils: A Review

**DOI:** 10.3390/ma17235977

**Published:** 2024-12-06

**Authors:** Marin Senila, Oana Cadar

**Affiliations:** INCDO-INOE 2000, Research Institute for Analytical Instrumentation, 67 Donath Street, 400293 Cluj-Napoca, Romania; marin.senila@icia.ro

**Keywords:** natural zeolite, toxic element, immobilization, contaminated soil

## Abstract

Soil contamination by toxic elements is a global problem, and the remediation of contaminated soils requires complex and time-consuming technology. Conventional methods of soil remediation are often inapplicable, so an intensive search is underway for innovative and environmentally friendly ways to clean up ecosystems. The use of amendments that stabilize the toxic elements in soil by reducing their mobility and bioavailability is one of the simplest and most cost-effective ways to remediate soil. This paper provides a summary of studies related to the use of composites based on natural zeolites and green materials for the immobilization of toxic elements in contaminated soils and highlights positive examples of returning land to agricultural use. The published literature on natural zeolites and their composites has shown that combinations of zeolite with biochar, chitosan and other clay minerals have beneficial synergistic effects on toxic element immobilization and soil quality. The effects of zeolite properties, different combinations, application rates, or incubation periods on toxic elements immobilization were tested in laboratory scale or field experiments, whereas the mobility of toxic elements in soil was evaluated by chemical extractions of toxic elements transferred to the plants. This review highlights the excellent potential of natural zeolites to be used as single or combined sustainable green materials to solve environmental pollution problems related to the presence of toxic elements.

## 1. Introduction

Soil is an essential, non-renewable natural resource that provides essential goods such as food, feed, fiber, and other resources for a circular bioeconomy [1]. Unfortunately, soil is a major reservoir or transport medium for various contaminants such as toxic elements, which are persistent contaminants because they cannot be degraded or destroyed by chemical or biological processes, and thus affect the biota through the food chain [2,3,4]. According to the European Environment Agency, more than 35% of European soils are contaminated with heavy metals [5]. The accumulation of toxic element trace metals in soils has the potential to impair soil functions (i.e., soil fertility and soil microbial activity and biodiversity), cause plant toxicity and enter the food chain with serious implications for human and animal health [6,7,8]. Worldwide, more than 10 million sites covering more than 20 million hectares of land with contaminated soils have been reported, of which more than 50% are contaminated with heavy metals and/or metalloids [2].

Toxic elements are a large group of elements with densities greater than 5 g/cm^3^ and high relative atomic weights that exhibit metallic properties such as ductility, malleability, cation stability, conductivity and ligand specificity [9]. In addition, there are elements that are highly toxic without biological functions in plants and can be classified as metals (Cd, Pb, Ag, Sr, Cr), metalloids (such as As and Sb) and non-metals (e.g., Sb). Toxic elements such as Cu, Fe, Mn, Mo, Ni, and Zn are known to be beneficial to biological systems but can become toxic when their concentrations exceed critical levels [10]. Moreover, the adverse environmental effects of polymetallic contamination are more complex and severe than those caused by single toxic elements due to their interactions, their reactions with the soil interface, the heterogeneity of the soil’s physical and chemical properties, etc. [11,12]. Multiple health effects are associated with exposure to heavy metals and metalloids including gastrointestinal, renal and bone dysfunction, neurobehavioral and developmental disorders, birth defects, skin lesions, immune system dysfunction, vascular damage, and tumor formation [13,14]. In addition, concurrent exposure to two or more metals may result in cumulative effects [15].

The risks posed by contaminants to humans and the environment depend not only on their total concentration, but also on the mobility of their reactions with the soil interface, the heterogeneity of the soil’s physical and chemical properties, etc. [16]. Depending on various factors, i.e., water, organic matter and clay content, temperature, pH, particle size, texture, nature of toxic elements and soil components, etc., the soil can adsorb, oxidize, reduce, exchange, catalyze or precipitate the metal ions [17]. In general, the soil-element composition and element ion concentration in soils are contingent on the mobility, solubility, and toxicity of toxic elements. Moreover, the availability of toxic elements in soils generally depends on the density and surface area of the soil and the ability to complex with ligands [10,18,19].

Toxic elements in soil come from both natural and anthropogenic sources. Natural sources include the weathering of metal-bearing rocks by rainwater and volcanic eruptions, while the anthropogenic sources include industrial activities (e.g., mining, smelting, petrochemicals, pigment production, batteries), domestic and municipal wastes, vehicle exhaust, and agrochemicals [20]. The major sources in the agricultural sector can be categorized as fertilizers, pesticides, livestock manure, and wastewater [21]. Toxic elements from polluted soils enter the food chain and contaminate feed and food, thereby endangering human and animal health. Thus, controlling and removing toxic elements from the environment has become a major challenge and requires radical and practical solutions to diminish the risks as much as possible [22,23].

Natural zeolites with outstanding ion exchange and sorption properties have gained worldwide recognition as cost-effective adsorbents that can be modified to remove/immobilize toxic elements from various environmental media, thereby reducing their uptake by plants and limiting food contamination [24,25,26,27]. In recent years, the research on the immobilization of metals in soil using zeolites and zeolite composites has flourished, resulting in new insights into the immobilization mechanisms, both at the laboratory scale and in field experiments [26,27,28,29,30]. In addition, to enhance the ability of natural zeolites to immobilize toxic elements in contaminated soils or to increase agricultural soil productivity, their combination with other sustainable materials has been reported [21,31,32,33,34,35]. To the best of our knowledge, to date, there is no single comprehensive review article that focuses on the existing information on this important topic. To fill this gap, the aim of this paper was to review the studies related to the use of natural zeolites and their composites or combinations with other sustainable green materials as attractive candidates for the immobilization of toxic elements in contaminated soils for their restoration in the agricultural circuit.

## 2. Amendments for Immobilization of Toxic Elements in Contaminated Soils

Various technical, physicochemical, agricultural engineering, and biological techniques have been developed to efficiently minimize the mobility and bioavailability of heavy metals and metalloids from soils through complexation, precipitation, adsorption, or ion exchange [1,22,23,36]. The stabilization technologies provide many benefits such as wide range of application, short remediation time, good remediation effect, low energy consumption, low engineering application cost, and minimal risk of additional environmental pollution [37,38,39].

The in situ and ex situ technologies used to treat contaminated sites are generally costly, environmentally unsafe and in many cases not feasible in practice [16,40,41]. The physicochemical method is a process that converts toxic elements into less bioavailable forms, but it can diminish the soil productivity and alter the chemical and physical structure of soils [18,42]. In recent years, the use of innovative approaches such as modified magnetic materials to address environmental problems (especially from aqueous effluents) has received considerable interest. Considering the diverse magnetic susceptibility range of the soil particles, the ferromagnetic materials can be separated by applying a low magnetic field, while the paramagnetic materials can be separated by applying a high magnetic field [36]. The biological method of removing metals from contaminated soils, i.e., phytoremediation, has emerged as a promising soil remediation technique that can readily absorb heavy metals and has proven to be an effective and economical technique. However, each plant responds differently to the uptake of specific toxic elements, and there are few plants that can treat all toxic elements at once. The application of phytoremediation methods in contaminated areas is a lengthy process because plants differ in their ability to extract and stabilize contaminants [43].

Traditional remediation technologies are expensive, making them practical for use on small areas of accidental contamination [1,43,44,45]. As a result, numerous alternatives that are considered to be less intrusive and more economical have been investigated. Of these, soil amendments can be used to increase the efficiency of phytoremediation and achieve the expected results [7,46]. Agricultural engineering methods include topsoil replacement or deep plowing. Various soil amendments such as biochar, lime, phosphate, fly ash, kaolinite, bentonite, steel slag, red mud, and zeolites have been shown to improve contaminated soils by immobilizing toxic metals [47,48].

For larger areas with cultivated land and historical contamination that has occurred over a long period of time, cost-effective and efficient alternative methods must be identified to minimize the adverse effects of contamination [49]. In situ immobilization of toxic elements offers a promising alternative to conventional remediation methods for reducing the environmental health risks posed by soil contamination with toxic elements. Immobilization techniques are based on the concept that reducing the levels of mobile forms, either by adsorption, complexation, or precipitation reactions, will make toxic elements less accessible for uptake by plants, cause leaching into groundwater, and result in consumption by animals and humans [50]. The addition of immobilizing amendments is a cost-effective and efficient approach to remediating contaminated soils, although it may not reduce overall contaminant levels. In situ soil remediation methods reduce the solubility and availability of toxic elements to plants through chemical stabilization using non-toxic materials [2]. Compared to excavation, immobilization is less costly and could offer a long-term remediation solution [51].

Various natural and organic materials derived from agricultural and industrial activities are often used to immobilize metals. Clay minerals, Al, Fe, and Mn oxides as well as hydroxides and zeolites, are natural materials known for their excellent sorption capacity [52]. Furthermore, the high organic matter content and improved biochemical properties of contaminated soils can be attributed to organic materials such as manure, compost, biochar, and biosolids, which help to reduce the presence of toxic elements in the soil [53]. Metal availability can vary due to various environmental factors and soil processes such as leaching, weathering, aging, acidification, and redox reactions. The durability of immobilized TE compounds, as well as the environmental impact and cost effectiveness of the amendments, should also be considered prior to application [54]. In addition, methods for immobilizing contaminants in situ must consider the durability of the soil treatment and its ability to reduce exposure levels [18].

## 3. Type of Natural Zeolites and Their Main Properties

Zeolites are a group of minerals with regular, defined crystalline structures comprising pores ranging from macropores to mesopores with excellent properties such as ion exchange, adsorption and dehydration [28,55]. They occur in sedimentary rocks of different ages and lithologies and belong to the authigenic silicate minerals. Many types of natural zeolites have been discovered in sedimentary deposits, the most common being clinoptilolite, heulandite, phillipsite, laumontite, and analcime, accompanied by less common types of zeolites such as modernite, chabazite, natrolite, erionite, or wairakite [56,57]. The main sources of natural zeolites are located in Slovakia, the United States, Greece, Bulgaria and Italy [58]. It is noteworthy that there are differences in sorption properties and capacities for toxic elements among natural zeolites from different geological sources, indicating that natural zeolites worldwide have different characteristics. They are mostly found in glass-rich volcaniclastic quarries, with volcanic glass being the main precursor of natural zeolites. The structure of zeolites is characterized by three main components: the aluminosilicate framework, molecules of zeolitic water, and major exchangeable cations. This group of minerals contains more than 50 species that have been identified [59]. Natural zeolites are classified according to their morphological characteristics, crystal structure, chemical composition, effective pore diameter, etc. The main types of natural zeolites are presented in Table 1 [56].

**Figure 1:**
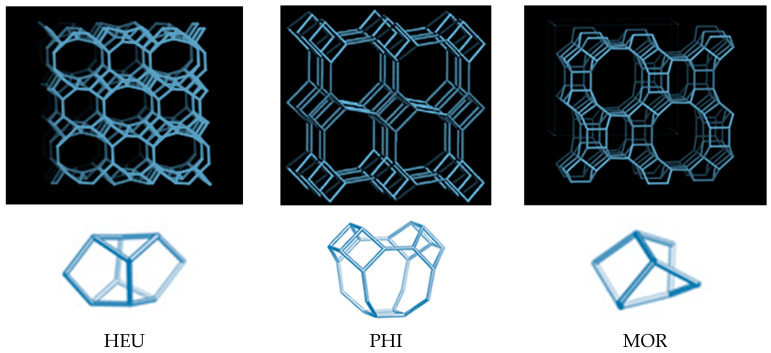
Composite building units and the mineral frameworks of selected zeolites (Structure Commission, 2020) [62].

The primary structure of the zeolite framework is a tetrahedron, built from Si or Al atoms, with oxygen atoms at the apices. Connected tetrahedra form cuboctahedra which are the basic cells of zeolite [60]. The composite building units and the mineral frameworks of three of the most widespread natural zeolites (heulandite—HEU, phillipsite (PHI) and mordenite—MOR) are shown in Figure 1 [61,62].

The Si/Al ratio is an important characteristic of zeolites. The isomorphic replacement of Si^4+^ by Al^3+^ explains the negative charge of the whole framework [63]. This negative charge is compensated by exchangeable alkaline and alkaline-earth cations, which are located together with water inside the aluminosilicate framework [57]. The Si/Al ratio has a direct relationship with thermal stability but an inverse relationship with the cation content. Based on the Si/Al ratio, the zeolites are classified into low (1.0–1.5), intermediate (2–5) and high (10-several thousands) Si/Al ratio zeolites [64]. Due to this cage-type structure and the presence of exchangeable cations and water molecules inside the cages, zeolites can switch substances with the external environment, which is their noteworthy characteristic [26,65].

Natural zeolites are environmentally friendly and low-cost materials with good adsorption and ion exchange properties owing to their special structure [62]. They have sorption and ion exchange properties that can be used to selectively retain cations of heavy metals from aqueous solutions, including from soil solutions [66]. In this way, they reduce the mobility of heavy metals in soils and their availability to biota. The adsorption properties of natural zeolites are unique because they act as molecular sieves, allowing small molecules to pass through the inlet channels and be adsorbed in the dehydrated channels and central cavities, while preventing the passage of large molecules [67]. The ability of zeolites to exchange cations is expressed quantitatively as the cation exchange capacity (CEC), measured in mmol/g zeolite. Depending on the characteristics of the zeolite, the CEC can be in the range of 1–4 mmol/g values higher than those of most other adsorbents [66,68]. Cation exchange behavior and selectivity depend on various parameters such as ambient temperature, charge density on the anionic framework, ion valence, electrolyte concentration, cation framework topology, ion size and shape, etc. [67]. Besides, due to its excellent CEC, faujasite is also a widely used zeolite for the adsorption of toxic elements from aqueous solutions [66].

**Figure 2:**
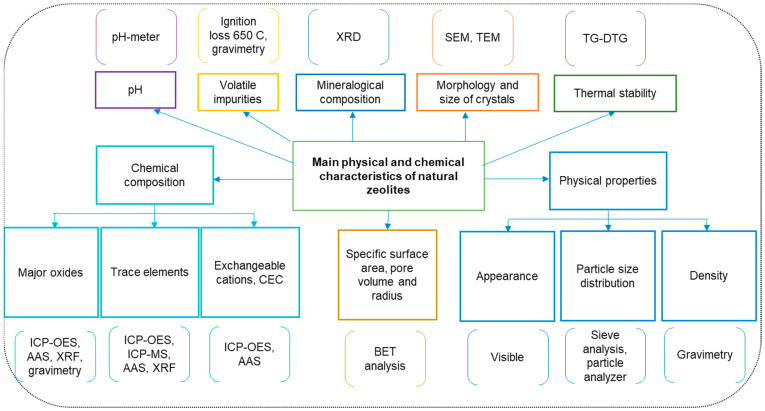
Commonly determined parameters for zeolites characterization.

Natural zeolites’ properties which are usually determined to assess their characteristics are chemical composition, CEC, mineralogy, morphology, size of crystals in zeolites, pore volume, specific area, and thermogravimetric behavior. The chemical composition refers to major oxides and trace elements and can be determined by zeolite sample digestion, measuring the elements by a spectrometric method (inductively coupled plasma optical emission spectrometry—ICP-OES, atomic absorption spectroscopy—AAS, and inductively coupled plasma mass spectrometry—ICP-MS), calculating the oxides using stoichiometry, and taking into account the atomic and molecular masses [69]. Because Si is difficult to dissolve, the SiO_2_ is typically determined by gravimetry. The CEC can be calculated from the measured concentrations of the extractable major cations (K^+^, Na^+^, Ca^2+^, and Mg^2+^) using a spectrometric method, after their replacement by ammonium ions by immersion in a solution containing this cation [70]. The mineralogical composition is determined by registering X-ray diffraction (XRD) patterns, whereas the size and morphology of zeolite crystals are evaluated using scanning electron microscopy (SEM) and transmission electron microscopy (TEM). Total surface area, total pore volume and pore radius, which are important parameters for the adsorption process, can be obtained from N_2_ adsorption–desorption isotherms using a Brunauer–Emmett–Teller (BET) instrument. Thermogravimetric-differential thermal analysis (TG-DTG) provides information about the thermal behavior of zeolites and can also differentiate some types of zeolites (e.g., clinoptilolite and heulandite) [71]. Figure 2 presents the main physicochemical parameters that are typically analyzed to characterize zeolites.

## 4. Application of Composites Based on Natural Zeolites and Green Materials in the Immobilization of Toxic Elements in Contaminated Soil

Stabilization of toxic elements with natural zeolites or zeolite composites is a viable approach for remediation of soils contaminated with toxic elements because they reduce the bioavailability and migration of toxic elements in soil, groundwater, or the food chain through ion exchange, complexation, adsorption, and precipitation [72]. These methods are economical, easy to use, require a short time for stabilization and have a reduced risk of secondary contamination [2,73]. Moreover, the addition of amendments into the soil is an efficient method for the remediation of soils contaminated with toxic elements due to its low soil disturbance [74]. Currently, amendments are generally classified into inorganic (lime, clay minerals, zeolites, industrial waste resins), organic (biochar, peat, straw, and compost), and inorganic–organic mixed with other material amendments in a certain proportion. Combined applications of different amendments have been shown to be effective and sometimes more beneficial than single applications in actual agricultural practice. The combined application of organic and inorganic amendments is expected to be the most effective agronomic method for remediation of soil contamination in the long term because of the combined benefits of increasing soil fertility and soil structure and reducing the bioavailability of toxic elements in the soil [32,75]. In addition to stabilizing toxic elements, the addition of natural zeolites can improve soil physical properties, does not significantly alter soil pH, increases soil water and nutrient retention capacity, and can act as a carrier for slow-release nutrients and pesticides [76].

### 4.1. Biochar

Biochar is produced by the thermochemical fragmentation of natural biomass in a very low oxygen environment. It has a porous carbonaceous structure, and because it is produced from biomass residues, it is considered as a green material and has gained worldwide acceptance. Several approaches such as pyrolysis, gasification, hydrothermal carbonization, torrefaction, and microwave heating are used to produce biochar [34,77,78]. Many types of organic wastes such as manure, wood, wheat husk, rice straw, corncob, bamboo, grass, etc., have been used to produce biochar [29].

As a soil amendment, biochar helps to improve soil fertility, enhance crop production, sequester carbon, and immobilize toxic elements in polluted soils due to its high surface area, cation exchange capacity, porosity, and abundance of surface functional groups. Due to its high stability, biochar can remain in the soil and function for long periods of time, improving nutrient and water retention, regulating the soil pH and increasing the immobilization of toxic elements [31,79,80]. The mechanisms involved in the decontamination of soil polluted with toxic elements using biochar comprises surface adsorption and precipitation or complexation mechanisms. The adsorption capacity of biochar is also related to soil characteristics, including cation exchange capacity, functional groups, or pore size distribution [81]. It has also been reported that biochar added to sewage sludge reduces the bioavailability of heavy metals and the nitrogen loss in the resulting compost [82]. Similar effects of nitrogen conservation in waste digestate during composting have been reported when zeolite is used as an amendment. The effect of adding zeolite, biochar and their mixture on nitrogen conservation and organic matter transformation during composting of pig manure was compared [83,84]. The results showed that all amendments improved the degradation of organic matter and helped to avoid nitrogen loss, but the combination of zeolite and biochar provided the best results during manure composting.

Biochar and natural zeolites have demonstrated remarkable potential for soil remediation, providing substantial environmental health and soil management benefits while successfully converting degraded soils to arable land [85]. Biochar creates a stable carbon structure that allows for the long-term stabilization of cadmium (Cd), while zeolite enhances adsorption potential and allows for ion exchange. This synergistic effect can enhance soil quality and mitigate the environmental risks associated with Cd contamination [86]. However, the natural zeolite was found to be a more effective soil amendment than biochar in the soil remediation process. Glab et al. reported no interaction effect of the zeolite and biochar combination, probably due to the type of species used, i.e., perennial grass species [22]. The application rate and mixing ratio of biochar and natural zeolite have a significant effect on the soil properties that determine the fractions of the toxic element species in the soil, and consequently their uptake by plants. Moreover, this combined application significantly enhances plant antioxidant enzyme activity, soil enzyme activity, and growth following the application of amendments that produced biochar from wheat straw, corn straw, licorice root pulp, rice husk, and sheep manure [32,81].

Feng et al. investigated the effect of combined application of biochar (0, 5, 10 and 20 g·kg^−1^) and nano-zeolite (0, 5, 10 and 20 g·kg^−1^) on Cd immobilization in contaminated soil (5 mg·kg^−1^ Cd) and promotion of Pak Choi growth using pot experiments [32]. When 20 g·kg^−1^ biochar and 10 g·kg^−1^ nano-zeolite were applied, the residual Cd in the soil increased by 214% as compared to the control. The combined application diminished the percentage of exchangeable Cd up to 44.8%, due to the increase in pH and soil organic matter content in Cd-contaminated soil, and consequently suppressed the Cd uptake by Pak Choi [81]. The zeolite–biochar composite prepared by mixing pyrolyzed zeolite and biochar in 7:3 mass ratio showed a 90% higher phosphorus removal in aqueous solutions compared to biochar and a maximum adsorption capacity of 2.41 mg/g [87].

Biochar single and biochar combined with a zeolite (12% biochar, and 10, 15 and 30% zeolite) were used as amendments to dewatered sewage sludge to improve the composting process and the value of the final product. A dosage of biochar–zeolite of 12% + 15% (dry weight basis) showed higher water-soluble and total macro-nutrients during composting under the 56 days of composting conditions and improved the yield of biomass (1.41 ± 0.12 g/pot) produced by Chinese cabbage (*Brassica rapa chinensis* L.) at 150 kg ha^−1^ [88]. Zheng et al. [33] conducted a 90-day incubation study to investigate the effect of rice husk biochar, natural zeolite and their combination in reducing the bioavailability of As, Cd, Pb and W in soil. The optimal amendment for soil remediation was found to be biochar–zeolite combination treatment, which reduced the total bioavailability toxicity from 335.5 to 143.4, but the combination of amendments has a lower stabilization rate for W than when using only zeolite. Increases in cation exchange capacity and pH were also observed in the amended soils [33].

Biochar obtained from poplar wood and char obtained from a mixture of poplar wood, grass, tires, cardboard, and plastic added to soil were added to contaminated soil and the influence on the uptake of Pb, Ni, and Cd by potatoes was evaluated. In this study, natural zeolite was tested as a reference for reducing metal uptake. The authors reported that biochar produced from poplar wood alone was more efficient in reducing metal uptake than biochar produced from a mixture of other wastes. Although natural zeolite also reduced the uptake of toxic metals by potatoes, it had a negative effect on potato growth [89]. Biochar produced from tobacco stalks and applied to soil reduced the bioavailability and transfer of heavy metals (Cd, Cu, Ni, and Pb) to tobacco, using a two-year field experiment with five biochar rates (0, 5, 15, 20, and 40 Mg ha^−1^) conducted in southwestern China on yellow-brown (pH 5.32) and yellow (pH 6.39) soils. The available Cd content increased with the biochar rate when the rate exceeded 15 Mg ha^−1^ in the acidic yellow-brown soil, and only at the rate of 40 Mg ha^−1^ in the neutral yellow soil. In all cases, the accumulation of Cu, Ni and Pb in tobacco decreased with the increasing biochar application rate [90].

Guo et al. [91] investigated biochar and zeolite as substrates to remove nutrients and As in biogas slurry as a technical support for the purification of As-containing biogas slurry in scalable breeding. The addition of biochar and zeolite increased the enrichment of As by sediments, water spinach and purple *Pennisetum sinese*, and decreased the absorption of As in green *Pennisetum sinese* [91]. In a similar study, the zeolite (clinoptilolite) and biochar enriched with recovered nutrients were used as soil fertilizers at two levels of N application (15 and 45 mg N per pot) [92]. It has been reported that zeolite (5%) and biochar (5%) added to agricultural waste altered the diversity of fungal and bacterial populations and influenced the composting process [93]. Hydrothermally modified natural zeolite added to food waste at 10% and 15% also improved the composting process [94]. While in many cases mixtures of zeolite and biochar have been used, Mosa et al. [95] prepared a composite material using biochar as a support for natural zeolite or recovery and recycling of aqueous phosphate and humate. In this regard, the chopped (0.5–1 mm size) water hyacinth plants were dried and mixed with a suspension of crushed zeolite in deionized water. After drying, the mixture was slowly pyrolyzed at 450 °C to obtain the biochar–zeolite composite.

Rahimi et al. [86] investigated the immobilization of Cd in contaminated calcareous soils (0, 75, and 150 mg/kg soil) using two amendments including biochar derived from grape pruning residues and natural zeolite (0, 1, and 4%) using a 16-week incubation period. The combination of biochar and zeolite reduced active Cd and its mobility in the soil due to increasing organic and carbonate fractions and diminished the amount of Cd extracted by DTPA (82.436 mg/kg) and EDTA (115.605 mg/kg). Cd content associated with organic matter showed the highest accumulation at a 4% biochar application rate. This research highlighted the effectiveness of organic amendments in immobilizing metals due to their significant cation exchange capacity and organic carbon content, surpassing the performance of mineral amendments.

Li et al. [21] investigated the synergistic effects of zeolite–biochar composite on the remediation of soil contaminated with Cd and Pb. The authors reported remediation efficiencies of 92.8% (Pb) and 92.9% (Cd), respectively, in stems under optimal conditions when using the composite material, along with the significant enhancement in the acidity and nutrient deficiency conditions of the red soil used. The long-term performance of natural zeolite (clinoptilolite) and biochar as low-cost adsorbents was evaluated to improve the treatment of leachate from constructed wetlands. The amendment with 10% (*v*/*v*) zeolite was found to increase nitrification rates by up to 93%, while 13% (*v*/*v*) biochar improved the removal of organic matter (up to 44%), microbial activity and plant growth, as well as heavy metal removal by adsorption [96]. Zhao et al. [34] prepared blocky recyclable biochar–zeolite composites by pyrolyzing a mixture of 50% zeolite and 50% feedstock at 400 °C, followed by their activation by its impregnation in a NaOH solution. The initial and NaOH-activated composites were found to reduce the bioavailability of Cd in soil by 59.70% and 68.54%, respectively. The composites studied also showed improved mechanical strength by losing only 4.06% and 5.40% under simulating mechanical sieving conditions, making them more suitable for soil application [97]. In general, the combination of biochar and zeolite was most effective in reducing metal bioavailability when compared to the application of each amendment individually. Thus, the use of biochar and natural zeolites in soil remediation is a promising tactic to improve soil quality and promote environmental sustainability.

### 4.2. Chitosan

One potential “green” metal chelator is chitosan, which is the second most abundant natural biopolymer and is obtained by the deacetylation of chitin found in seafood waste [98]. Currently, chitosan is being widely regarded as an excellent natural adsorbent due to the presence of amine (-NH_2_) and reactive hydroxyl (-OH) functional groups that act as chelating sites for metal ions through ion exchange, adsorption, and complexation [8,99].

The combination of natural zeolites with chitosan is possible due to the hydrogen bonding between the hydroxyl and amino groups of chitosan and between the hydroxyl groups of the zeolite surface. The silicon hydroxyl groups of zeolites can also react with the carbon hydroxyl groups of chitosan, resulting in a very stable bond [100]. In addition, loading with chitosan can alter the surface properties of the zeolite, increasing hydrophilicity, increasing specific surface area, and providing more binding sites for toxic elements [73]. However, to the best of our knowledge, the evaluation of their stabilization effects after their application in soil is lacking.

Considering the poor mechanical properties of chitosan and the need to obtain an interpenetrating network structure to enable the stabilization of toxic elements in soil, Ma et al. [73] prepared an economical, efficient, and environmentally friendly material using a four-step method, i.e., the zeolite pretreatment with sodium chloride, chitosan modification, zeolite loading with modified chitosan, and calcium silicate modification. The modification process improved the stabilization efficiency of the zeolite material to simultaneously stabilize multiple, available toxic elements in the soil and thereby reduce the risk of leaching and their accessibility to plants. This composite was found to reduce the weak acid-extractable Pb, Cd and Zn fractions in soils from a chemical industrial park in China by 21, 10 and 19%, respectively, resulting in a stabilization efficiency of 90.1%, 97.7%, and 88.6% 56 days after amendment [73].

Wen and Zeng [101] used chitosan-coated zeolite to reduce the leachability of heavy metals from soil mixed with dredged contaminated sediment. Natural zeolite from China (particle size 0.5–1.0 mm) was treated with 1 M HNO_3_ solution, mixed with chitosan in 5% acetic acid at 1:1 mass ratio to obtain a hydrogel, followed by precipitation with 0.5 M NaOH solution. Pot experiments were performed by mixing sediment or sediment + soil combination with natural zeolite or with chitosan-coated zeolite. The BCR sequential extraction scheme was applied to evaluate the fractionation of Cd, Zn, Pb, Cu, Ni and Cr into a acid-exchangeable fraction, reducible fraction, oxidizable fraction and residual fraction. Considering the acid-exchangeable fraction as the most mobile, it was reported that chitosan-coated zeolite significantly reduced the Cd mobility in the sediment–soil mixture (7%) and dredged sediment (8%). Chitosan-coated zeolite also reduced the toxicity response of *E. fetida* cultivated in low and high Cd-spiked sediment–soil mixtures by decreasing the tissue Cd content and earthworm mortality rate.

The chitosan–zeolite composite material, which was obtained by mixing chitosan and zeolite in a mass ratio of 1:1 in 5% acetic acid and cross-linked with epichlorohydrin showed an excellent maximum adsorption capacity of 275 mg g^−1^ for Pb^2+^ ions from a 1000 mg L^−1^ Pb aqueous solution, at pH = 4.5 and chitosan–zeolite composite dose of 5 g/L [102]. The theoretical simulations carried out using Monte Carlo simulations indicated that Pb displayed a noticeable preference for adsorption on the chitosan–zeolite composite adsorbent surface. Thus, the chitosan–zeolite material has an excellent potential to be used for Pb immobilization in soil solution.

### 4.3. Natural Minerals

Due to their abundance and high specific surface area, natural materials such as clay minerals (e.g., zeolite, bentonite, kaolinite) are environmentally friendly and financially affordable adsorptive materials for removing heavy metals from soils while providing suitable conditions for plant growth [103]. Bentonite characterized by two layers of Si^4+^ ions and one layer of Al^3+^ ions, is considered one of the most promising candidates for decontamination due to its high porosity, cation exchange capacity, low metal solubility, and ability to adsorb both organic and inorganic ions [104]. Moreover, the application of bentonite in the soil improves the efficiency of water use, provides a continuous supply of essential minerals to maintain plant growth during periods of deficiency, and increases plant resistance to abiotic stress [29].

The adsorption capacity of natural zeolites can be enhanced by chemical surface modification with Mn and Fe oxides. In addition, the addition of these oxides to soils increases the immobility of heavy metals, leading to the remediation of heavy metal contaminated soils [105]. In this regard, goethite is a common soil mineral and one of the most thermodynamically stable Fe oxides. It has excellent potential as an adsorbent to enhance pollutant immobilization because of its large surface area and specific surface active sites [106]. Argiri et al. used zeolite, bentonite, goethite and two adsorbents consisting of zeolite (system I) and goethite (system II) as effective tools for Pb adsorption from a soil (18% sand, 48% clay and 34% loam) with a particle size less than 15 mm at a rate of 1 g/kg of soil and using wheat, maize and cotton for 40 days [107]. The soils containing goethite and zeolite–goethite (system II) in pots with maize and wheat showed the greatest reduction in available Pb (extractable in diethylenetriaminepentaacetic acid, DTPA). Compared to the control, the plant uptake of Pb was significantly reduced in all treated soils, especially in those treated with zeolite–goethite (system I) in pots with maize [107].

According to Afzal et al., the application of mesquite biochar in combination with zeolite and bentonite, in a 20:40:40 wt.% ratio, can be a low-cost and effective solution to immobilize lead in the soil, provide essential nutrients, and improve the physiochemical properties of the soil to promote plant growth [29]. The pot experiment was conducted in a greenhouse facility using soil artificially spiked with a solution level of Pb (600 mg/kg soil) and Zn (300 mg/kg soil). The obtained results revealed that the organo-mineral amendments improved the physiological and biochemical characteristics of maize crop, and simultaneously reduced the bioavailability and mobility of Pb and Zn in contaminated soils, thus reducing the metal accumulation by plants and enhancing the antioxidant system.

### 4.4. Various Materials

A composite material containing natural zeolite as support for nanoscale zero-valent iron was prepared and used to immobilize Sb in contaminated soil [108]. An application rate of 3% to the soil resulted in a 76% decrease in the leached concentration of Sb (from 1.32 mg L^−1^ to 0.31 mg L^−1^). Also, the mobile species of Sb in the soil decreased from 19.84 mg kg^−1^ to 0.71 mg kg^−1^, after 60 days of incubation, with an immobilization efficiency of about 90%. A zeolite-supported nanoscale zero-valent iron material was also prepared and found to be an efficient material for the adsorption of Cd, Pb, and As [109]. This material significantly immobilized Cd, Pb, and As in the soil at an application rate of 30 g kg^−1^.

The co-remediation effect of zeolite and humic acids on artificially Pb-contaminated garden soil, in terms of the Pb fraction of sequential extraction in the soil and the distribution of Pb in different parts of rape, was investigated using pot experiments [110]. The co-application of zeolite and humic acids reduced the available fraction of Pb, slightly increased the water-soluble fraction of Pb in the garden soil, compared with the application of single zeolite, particularly in the highly Pb-contaminated soil (≥1000 mg kg^−1^), and decreased the Pb content in the edible parts of rape (aerial parts) in the low Pb-contaminated soil, thus permitting the re-establishment of vegetation at contaminated sites [110].

## 5. Mechanism of Toxic Elements Immobilization by Composites Based on Natural Zeolites and Green Materials

**Figure 3:**
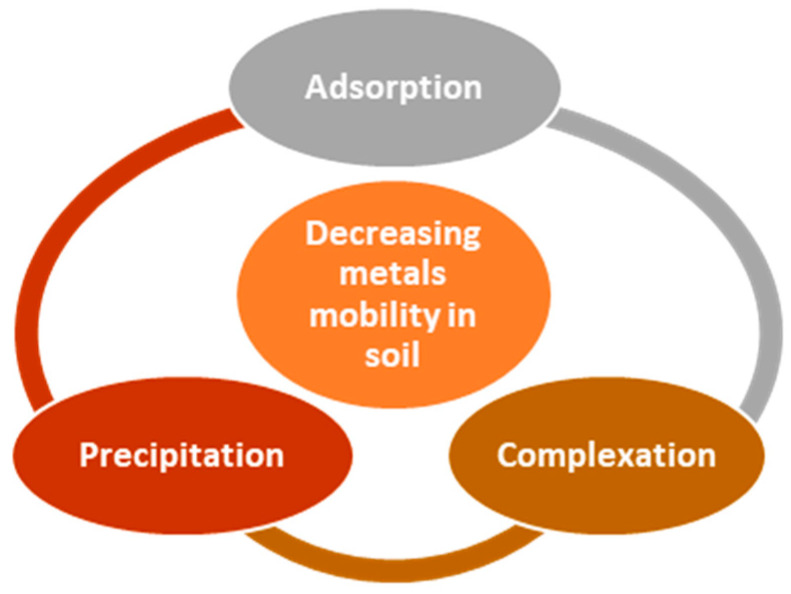
Processes that contribute to the decreasing of metal mobility in soils.

The amendments diminish the mobility, bioavailability and bioaccessibility of the toxic elements present in the soil by complexation, precipitation and adsorption, which is summarized in Figure 3 [32,74].

**Figure 4:**
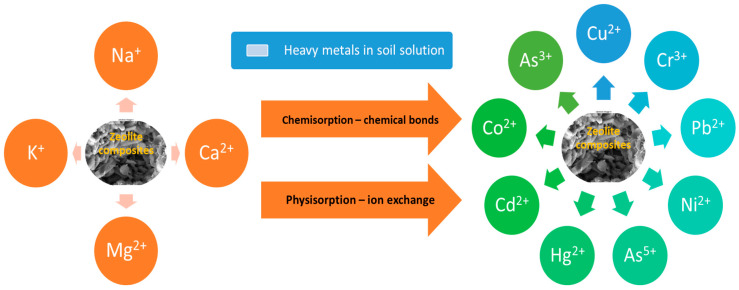
The adsorption mechanisms of metal mobility in soils by zeolite composites.

The adsorption mechanisms of zeolites can be classified mainly into chemisorption and physisorption, as presented in Figure 4.

Chemisorption implies the formation of chemical bonds between the zeolite surface and the adsorbed substance and is described by the Langmuir model [111]. Chemisorption is, generally, an irreversible process, which makes desorption more difficult. Physical adsorption is usually attributed to van der Waals forces and depends on the active surface and is mainly reversible under certain conditions [112]. Ion exchange is a process in which a counter ion from the zeolite surface is replaced by an equivalent number of moles of another counter ion to maintain the neutrality of the ion exchanger. In this process, the ions are physically adsorbed, with their inner hydration shell fully retained, and this adsorption occurs because of electrostatic or coulombic attraction.

The retention process of metal ions (M^z+^) on zeolites starts with the formation of complexes involving ion exchange reactions among M^z+^ and exchangeable cations from the zeolite framework (mainly Na^+^ and Ca^2+^) that pass into the solution [113]. The charge of the ions, their concentration in the solution and their size strongly influences the process of ion exchange. Ion exchange increases with the charge of the ions. Also, ions with a smaller hydrated diameter enter more easily into the pores of favoring the exchange process [114]. As the sorption process progresses, M^z+^ are fixed to the internal active sites of the zeolite structure, forming complexes of the inner sphere, which are more stable because of the resulting stronger covalent bond [68].

Shi et al. summarized that natural zeolites can basically lead to the immobilization of toxic elements in three ways: (i) zeolites dissolve, providing alkalinity to the acidic polluted soils causing the precipitation of insoluble phases. The resulting phases contain metals as major components or as minor components co-precipitated in hydroxides; (ii) the higher alkalinity and large surface area of natural zeolites favor metal sorption as the minerals become positively charged at low pH due to proton sorption and become negatively charged at high pH due to surface deprotonation of unsaturated bonds; and (iii) metal retention can occur at any pH due to cation exchange in natural zeolites [18,26,110]. Practically, the main mechanism of toxic element immobilization is a combination of the ion exchange properties of the natural zeolite and its ability to improve the soil pH. The immobilized toxic elements are fixed within the zeolite framework or precipitated as carbonates or oxides as the soil pH increases. The chemical fixation by natural zeolites could also be related to the degradation of toxic elements present in the contaminated soil because of its adsorption on the zeolite framework. The natural zeolite can fix the toxic elements in the mineral clay layer of the soil by diffusion and increase the pH of the soil for a longer period of time [28,115,116,117,118]. In addition, the remediation efficiency depends on the increase in metal sorption of the resulting soil–material mixture, on the dilution of the contaminant concentration when large doses of material are used, and on the surface precipitation/co-precipitation mechanism, which is controlled by insoluble/soluble products interacting with additional minerals [8,119].

For determining the chemical state of immobilized elements, several authors utilized instrumental methods such as extended X-ray absorption fine structure (EXAFS) and X-ray absorption near edge structure (XANES), two subcategories of X-ray absorption spectroscopy (XAS). XAS offers the essential tools required for analyzing the fine structures and oxidation states of elements. Additionally, it also supports studying the interaction mechanism of metals. XAS provides the atomic structure and valence of heavy metals present in soils or zeolite frameworks.

Sushkevich et al. [120] used wavelet transform analysis of EXAFS to perform a study of Cu species in Cu-exchanged zeolites with different frameworks, Si/Al ratios and Cu loadings, and found out that the zeolite topology affects the Cu species held in zeolites of different topologies. Pankin et al. [121] used XANES simulation and EXAFS wavelet transform for the identification of Cu-oxo species in Cu-modified zeolites. Chen et al. [122] investigated Cr(VI) immobilization in zero-valent iron-supported zeolite using EXAFS and XPS techniques. XANES/EXAFS spectroscopy was employed to assess the Cd speciation and distribution in contaminated soils and rice seeds. Based on the results of this technique, the authors showed that Cd occurred mainly as Cd(II)-O in the soil and crops [123]. Consequently, XANES/EXAFS results may provide essential information on metals’ transport routes from soils to crops and an explanation of a bioaccumulation mechanism of elements in crops.

## 6. Effects of Composites Based on Natural Zeolites and Green Materials on Toxic Elements in Soil

Heavy metals are known to have adverse effects on the environment and human health. In soil, the mobile fractions of heavy metals are bioavailable and can be transferred to the biota [124]. Compared with other soil remediation technologies, chemical immobilization has been widely used to remediate heavy metal-contaminated agricultural soils due to its high efficiency, low cost, and ease of operation. In this regard, the enhancement of chemical immobilization by the addition of soil amendments is a valuable alternative approach that can effectively reduce the bioavailability of toxic elements in soil by converting highly active fractions into stable fractions [35]. The use of large-area amendments in toxic element-contaminated soils increases plant growth, which may be related to their role in reducing the availability of toxic elements in the soil and, consequently, promoting soil microbial activity. Various synthetic and natural amendments can decrease the bioavailability and mobility of toxic elements from contaminated soils. However, the combined application of biochar and natural zeolites is also able to increase the microbial activity, soil productivity and plant growth, which is considered to be environmentally beneficial and cost effective for the remediation of toxic element-contaminated soils [125].

Laboratory-scale studies and field experiments have demonstrated the effectiveness of natural zeolites and their modified forms in reducing the concentration of toxic elements in plants [126]. Natural zeolites are well known for their ion exchange and adsorption properties, as well as being environmentally friendly, low cost, renewable, easily accessible, and available, which make them an excellent choice as adsorbent materials. The affinity of adsorbent materials for toxic elements can be increased by modifying their composition or by combining them with other materials. The modification of natural zeolites to increase their adsorptive capacity includes physical and chemical modification and the preparation of composites [127]. Physical modification is mainly achieved by thermal treatment. The efficiency of zeolites in reducing the transfer of toxic elements to the upper crops by stabilizing metals in polluted soils has also been studied in combination with other natural materials or by producing composites.

Table 2 summarizes several studies on the use of natural zeolites, composites or combinations of zeolites with other natural materials to obtain amendments and the effects on metal mobility in soil.

## 7. Influences of Soil Characteristics and Soil Types on Metal Immobilization

The mobility of heavy metals in soils is strongly influenced by soil characteristics and composition. The metal’s bioavailability in the soil is defined as its freely available fraction, not sequestered in the soil solid phase, which is mobile or easily mobilizable [131,132,133]. Consequently, the bioavailability of metals in soils depends not only on their overall abundances but also on their interactions with inorganic and organic soil components and the soil’s physical and chemical characteristics. The main soil characteristics that affect metal mobility and bioavailability include content of organic matter, clay content, soil pH, cation exchange capacity and redox potential [105].

Soil minerals and clay minerals are the main components of solid soil matrices. These mainly include silicate minerals, amorphous oxides, and crystalline oxides, which significantly influence metal mobility in soil, while organic matter accounts for about 1–10% of soil components [134]. Metals can fix the surfaces of clay minerals by different non-specific or specific adsorption processes. Specific adsorption is quite a strong process that arises at variable charge sites on mineral surfaces. In contrast, non-specific adsorption is weak and arises on mineral surfaces at permanently charged sites [135]. Organic matter in soil usually forms complexes with metals in soil and can raise the adsorption capacity for metals in soils, thus dropping their bioavailability in soils. Soil pH also plays a key role in controlling the mobility of metals in soil.

Generally, the availability and accumulation of metals in crops was found to be negatively correlated with pH. The increasing soil pH produces intensifications of the negative charge on the soil colloid surface which increases the adsorption capacity of the metal [136,137]. In particular, plant species transfer metals from soil into their tissues differently, even when planted on the same soils with same contamination levels [85].

Toxic elements such as Cu, Zn, Pb, Cd, As, Cr, and Hg were reported as the most widespread inorganic contaminants of soils [138]. The existence of these elements in increased contents in the soil has the effect of reducing crop production and resulting in their transfer into the food chain. Understanding their behavior between soil and other environmental compartments (water, biota) is highly necessary to choose the best remedial options. Specifically, the effect of added amendments to the soil for their immobilization was extensively studied. Clinoptilolite, the most abundant natural zeolite, was widely studied regarding the selectivity series for different cations. Zamzow et al. [139] reported the following series of selectivity: Pb > Cd > Cs > Cu > Co > Cr > Zn > Ni > Hg. Natural zeolite sampled from the Yagodnisky deposit showed the following order of selectivity Cu > Fe > Ni > Co [140]. In a study on Romanian thermally treated clinoptilolite-type zeolite, the following order was reported: Pb > Cr > Cu > Zn > Cd > Ni [71]. Given these findings, the affinity of natural adsorbents toward different toxic elements can differ depending on the adsorbent’s specific properties, thus these should be tested before application on soil.

## 8. Limitations

Over the years, there has been a growing interest in recognizing the use of natural zeolites in environmental remediation and agriculture because of public concern about the quality and sustainability of soil resources under intensive production systems.

Because the remediation of contaminated soils is an inherently interdisciplinary process, the associated discussion is extensive since it involves a variety of perspectives on monitoring approaches. The type, degree of contamination, characteristics of the soil, as well as regulatory requirements, play a critical role in determining the best strategy and appropriate remediation technology. In addition to the numerous studies reporting the advantages of using composites based on natural zeolites and green materials as amendments in soils contaminated with toxic elements, there is little information in the literature regarding the potential drawbacks of using natural zeolites on a long-term basis. The main drawbacks are related to the lack of information on (i) the long-term stability and persistence of natural zeolites in a complex system such as soil; in addition to climatic and environmental conditions, the type, size, cation exchange capacity, structural defects, type of cage structures and ability of ion-absorption of natural zeolites play a key role in their wide variation, (ii) the persistence of TE immobilization is not fully elucidated because the binding zeolite-TE is not well studied, (iii) long-term effect of natural zeolites on soil pH, and (iv) the possibility of releasing large amounts of elements such as Na and K via the exchange process [27,141,142]. The immobilization of toxic elements by natural zeolites and their composites is mainly governed by the pH value and cation exchange. Although several investigations have been carried out in recent years, comprehensive and effective studies are needed for future development stages to demonstrate which factor is more important in pot and field experiments.

The adsorption capacity of natural zeolites cannot be compared with that of synthetic materials, which have a tailored composition, structure and properties. However, this disadvantage can be offset by the low cost of natural zeolites and their widespread availability in large quantities around the world [26,143,144]. Moreover, when combined with other materials, the leaching of toxic elements from their use and the long-term effect on soil pH are additional limitations. For example, the use of fly ash, which has a very high pH, can cause a significant increase in soil alkalinity, thus negatively affecting both soil and plant ecosystems. However, the zeolitization process reduces this risk by immobilizing toxic elements derived from the raw waste material within the structure of the newly formed zeolite [145,146].

## 9. Conclusions

In this review, we have provided a detailed overview of the composites based on natural zeolites and green materials as excellent materials for the immobilization of toxic elements in contaminated soils. The properties of natural zeolites (i.e., adsorption, ion exchange, dehydration, and rehydration), as well as their low cost, environmental friendliness, regenerability, easy accessibility, and availability, make natural zeolite an excellent choice as a cost-effective adsorbent for immobilizing toxic elements from contaminated soils. Due to their properties, natural zeolites have been reported to treat contaminated soils by converting the mobile fractions of toxic elements into less bioavailable forms, thus reducing their migration in soil, groundwater or the food chain. Applications of natural zeolites in combination with other amendments have been shown to be more beneficial than single applications due to the combined benefits of reducing toxic element mobility and improving soil fertility. One of the most widely studied co-amended zeolites is biochar, a cost-effective and sustainable material that can be produced from many types of organic waste. Biochar improves soil fertility and crop production, sequesters carbon, and actively participates in the immobilization of toxic elements in soils. Another “green” material being studied as a metal chelator is chitosan, which is the second most abundant natural biopolymer. Its combination with the natural zeolites showed an excellent adsorption capacity of heavy metal ions. Many other studies have combined natural zeolites with other inorganic natural minerals such as clay minerals (bentonite, kaolinite, goethite). Therefore, people from countries with natural zeolite resources will benefit by reducing costs and pollution by using natural resources instead of using other types of toxic and/or synthetic adsorbents that produce various types of pollutants.

## Figures and Tables

**Table 1:** Major types of natural zeolites and their main properties.

Zeolite Type	Si/(Al + Fe^3+^) Ratio	Water Molecules	Main Exchangeable Cations
Clinoptilolite	4.0–5.6	3.5–4.0	Na^+^, Ca^2+^, K^+^
Heulandite	2.7–4.0	2.5–3.1	Ca^2+^, K^+^, Na^+^, Sr^2+^
Phillipsite	1.08–3.35	1.7–3.3	Ca, K^+^, Na^+^
Laumontite	1.9–2.4	2.0	Ca^2+^
Analcime	1.5–2.9	1.0–1.3	Na^+^
Modernite	4.1–5.7	3.0–3.5	Ca^2+^, Na^+^, K^+^
Chabazite	1.4–4.1	2.7–4.1	Ca^2+^, Na^+^
Natrolite	1.5	1.0	Na^+^
Erionite	2.6–3.8	3.0–3.5	Na^+^, K^+^, Ca^2+^
Stilbite	2.6–3.5	2.8–3.5	Na^+^, Ca^2+^
Wairakite	2.0	1.0	Ca^2+^

**Table 2:** Effect of amendments based on zeolites combined with other natural materials on metals in soil.

Type of Zeolites and Their Modification	Combination of Zeolites	Mode of Application on Soil	Effect	References
Analcime zeolite obtained from red soil	A composite material containing zeolite and biochar from rice straw.	Composite materials of zeolite and biochar added to contaminated soil at amounts of 10, 20, and 30 g/kg.	Zeolite and biochar composite showed synergetic effect on soil remediation. The contents of TN, TOC, available P and available K in soil increased by 41.2%, 42%, 172.0%, and 878.0%. Pb and Cd in the plant stems diminished by 92.8% and 92.9%.	[21]
Zeolite	Application of organo-mineral amendments: biochar bentonite and zeolite alone and in combination (20% biochar, 80% bentonite or zeolite; 20% biochar, 40% zeolite, 40% bentonite).	Control soil and soil artificially spiked with Pb and Zn, and then kept in the dark for 1 month. Soil was used for growing maize in greenhouse pot experiments.	The amendments reduced the mobile fractions of metals in soil and reduced the transfer of Pb and Zn in maize roots by 24–59% and 42–68% and leaves by 19–60% and 43–75%.	[29]
Natural zeolite from Iran with grain size < 0.5 mm	Natural zeolite added to soil with biochar produced from corn straw, wheat straw, rice husk, licorice root pulp, and sheep manure.	Natural zeolite applied at 0, 3 and 6% (*w*/*w*) combined with biochar at 3% (*w*/*w*).	Combination of sheep manure biochar with zeolite provided the best results to decrease Cd mobility in soil. EDTA-extractable Cd decreased with 54.2%.	[81]
Natural zeolite	Natural zeolite, biochar and their mixture used as amendments for soil.	Ninety-day incubation in pot experiments for an application rate of 5% amendment to soil.	Bioavailability of As, Cd, Pb, and W decreased by 57.4, 62.7, 56.4, and 22.5%, respectively, following amendments application to soil.	[33]
Natural zeolite powder	Zeolite–biochar composite obtained from co-pyrolysis of 50% zeolite with 50% feedstock, then activated by NaOH.	Composites mixed with soil at 5% mass, batch experiments.	Bioavailability of Cd in the soil decreased by 59.70% and 68.54%, respectively, following zeolite–biochar composite application to soil.	[97]
Natural zeolite anzimit type	Natural zeolite alone.	Zeolite application at increasing amounts of 0, 5, 10, and 15 g kg^−1^ of soil contaminated with Cd. Soil was used to grow corn.	Cd availability was significantly reduced in all soils amended by zeolites, until 86.84%. Growth characteristics of corn improved and N, P, and K in leaf increased by 71.20%, 47.01%, and 20.19%	[128]
Clinoptilolite type natural zeolite from Turkey	Zeolites and biochar separately added for comparison.	Zeolite at 5%, 10%, and 20% and biochar at 1%, 2%, and 4% were added to soils irrigated with wastewater.	Biochar and zeolite addition to the soils reduced the metals mobility. The concentrations of heavy metals in the leaching waters decreased.	[129]
Natural zeolite from Bulgaria	Zeolite, zeolite–biochar composite, and vermicompost added to degraded soil.	One percent of soil amendments were mixed with soils: Eutric Cambisol (Poland) and Epicalcic Cher nozem (Bulgaria).	Zeolitic and vermicompost materials enhanced Cd adsorption. The amounts of Cd adsorbed were 1.04 mg/g and 2.97 mg/g in the two soils from a solution with an initial Cd concentration of 50 mg/L.	[130]
